# The Burden and Benefits of Knowledge: Ethical Considerations Surrounding Population-Based Newborn Genome Screening for Hearing

**DOI:** 10.3390/ijns8020036

**Published:** 2022-05-27

**Authors:** Calli O. Mitchell, Greysha Rivera-Cruz, Matthew Hoi Kin Chau, Zirui Dong, Kwong Wai Choy, Jun Shen, Sami Amr, Anne B. S. Giersch, Cynthia C. Morton

**Affiliations:** 1 Department of Obstetrics and Gynecology, Brigham and Women’s Hospital, Boston, MA 02115, USA; comitchell@bwh.harvard.edu; 2 Department of Pediatrics, Division of Genetics and Genomics, Boston Children’s Hospital, Boston, MA 02115, USA; greysha.rivera-cruz@childrens.harvard.edu; 3 Department of Obstetrics & Gynaecology, The Chinese University of Hong Kong, Hong Kong, China; matthewchau@cuhk.edu.hk (M.H.K.C.); elvisdong@cuhk.edu.hk (Z.D.); richardchoy@cuhk.edu.hk (K.W.C.); 4 Shenzhen Research Institute, The Chinese University of Hong Kong, Shenzhen 518172, China; 5 Hong Kong Hub of Paediatric Excellence, The Chinese University of Hong Kong, Hong Kong, China; 6 The Chinese University of Hong Kong-Baylor College of Medicine Joint Center for Medical Genetics, Hong Kong, China; 7 Department of Pathology, Brigham and Women’s Hospital, Boston, MA 02115, USA; jun.shen.98@gmail.com (J.S.); samr@bwh.harvard.edu (S.A.); agiersch@bwh.harvard.edu (A.B.S.G.); 8 Harvard Medical School, Boston, MA 02115, USA; 9 Laboratory for Molecular Medicine, Partners Healthcare Personalized Medicine, Cambridge, MA 02139, USA; 10 Broad Institute of MIT and Harvard, Cambridge, MA 02142, USA; 11 Manchester Centre for Audiology and Deafness, School of Health Sciences, University of Manchester, Manchester M13 9PL, UK

**Keywords:** genome sequencing, newborn hearing screening, newborn screening, newborn genome sequencing, incidental findings, secondary findings, carrier status

## Abstract

Recent advances in genomic sequencing technologies have expanded practitioners’ utilization of genetic information in a timely and efficient manner for an accurate diagnosis. With an ever-increasing resource of genomic data from progress in the interpretation of genome sequences, clinicians face decisions about how and when genomic information should be presented to families, and at what potential expense. Presently, there is limited knowledge or experience in establishing the value of implementing genome sequencing into newborn screening. Herein we provide insight into the complexities and the burden and benefits of knowledge resulting from genome sequencing of newborns.

## 1. Introduction

Since the 1960s, with initiation of the Guthrie card [1], newborn screening (NBS) has improved the lives of countless newborns and their families. The mandate of NBS is to identify treatable conditions in the newborn period that are not necessarily apparent at birth, but that could have life-long or fatal sequelae if untreated. A notable NBS advancement was the introduction of Newborn Hearing Screening (NBHS) in 1994 after endorsement by the Joint Committee on Infant Hearing [2]. This public health initiative makes possible early diagnosis and management for deaf and hard-of-hearing (DHH) babies, and better health outcomes for children and their families worldwide. Today, NBHS has been widely adopted in the United States with greater than 97% of newborns screened by one month of age [3].

Congenital deafness affects 1.7 of 1000 newborns [4] in the United States and is the most prevalent congenital sensory anomaly diagnosed in industrialized countries [5]. DHH is unparalleled in its heterogeneity with both genetic and environmental causes. With greater than 50% of congenital DHH having a genetic etiology, the importance of genetic testing in diagnosis and management of DHH individuals is paramount. Among genetic forms, both nonsyndromic and syndromic cases may result from variation in individual genes, and the same gene may be responsible for either dominant or recessive DHH. Over 400 types of syndromic DHH are recognized by the involvement of other organ systems in addition to the inner ear [6]. Nonsyndromic deafness is more prevalent than syndromic deafness and accounts for 70% of hereditary DHH [7].

## 2. Considering Health Outcomes for DHH Individuals

Given the auditory dominance of personal communication in the world, deafness has implications for an individual’s well-being on all socioecological levels, disrupting interpersonal interactions, relationships in community settings, and with society at large [8]. Due to inheritance patterns and environmental causes of hearing loss, greater than 90% of DHH individuals are born to hearing parents [9] who likely have limited knowledge surrounding deafness. Many factors influence communication among children and adolescents, including the degree of hearing loss. Studies have shown that DHH individuals may experience barriers which impact educational attainment, the likelihood of future employment, future earnings, use of healthcare systems, and life expectancy [10]; thus, access for all to early diagnosis and interventions are crucial for DHH health outcomes.

Shearer and co-authors provide promising evidence that a genetic etiology for hearing loss can influence the treatment and management of a child’s care [11]. In fact, research demonstrates the established benefits on speech performance after cochlear implantation of infants with *GJB2* or *SLC26A4* diagnoses [12,13]. The benefits of cochlear implantation for genetic etiologies including *OTOF*, *CACNA1D*, *CABP2*, *SLC17A8*, *DIAPH3*, *OPA1,* and *ROR1* have been illustrated [11]. Thus, a comprehensive approach to newborn hearing screening which includes genome sequencing (GS), physiologic hearing screening, and congenital cytomegalovirus (cCMV) testing [14] provides beneficence to DHH newborns and may influence development of future interventions or therapeutics.

## 3. Comprehensive Newborn Genome Sequencing: SEQuencing a Baby for an Optimal Outcome

SEQaBOO (SEQuencing a Baby for an Optimal Outcome), a research project initiated in Boston, Massachusetts [15], offers genome sequencing for newborns who are referred for diagnostic audiometry following physiologic screening. DHH is serving as a paradigm for integrating population-based genome screening into NBS at large. Recruitment is ongoing at three Harvard-affiliated hospitals: Brigham and Women’s, Boston Children’s, and Massachusetts Eye and Ear. SEQaBOO provides comprehensive genome sequencing and variant interpretation of DHH-associated genes, as well as optional (for parents only) ACMG secondary findings (SF) v 3.0 [16]. In collaboration with the Chinese University of Hong Kong, genome-wide copy number variant (CNV) analysis is assessed on all participants [17,18,19]. This analytic platform can also identify chromosomal aneuploidy, absence of heterozygosity, and chromosomal structural rearrangements including translocations. Through this method, balanced chromosomal translocations, sex chromosome aneuploidy, and pathogenic CNVs in genes associated with DHH have been detected. Such findings resulted in numerous protracted conversations surrounding the positive and negative ethical implications of disclosing incidental genomic findings from a research study that ultimately may not be related to the infant’s DHH phenotype.

SEQaBOO has proven the feasibility of implementing comprehensive genome sequencing into NBHS to facilitate earlier intervention and treatment options for DHH individuals. Similar studies have demonstrated the importance of genetic screening in the newborn period as a mechanism to improve health outcomes for DHH individuals [20,21,22,23,24,25]. For example, newborns with a positive genetic finding may pass physiologic NBHS as frequently as 50% of the time yet are diagnosed as DHH later in childhood [15]. A population-based study of this capacity also has the potential to identify novel genomic variants contributing to DHH and other heritable disorders.

## 4. Ethical Dilemmas in Real Time

Given the comprehensive nature of SEQaBOO, various ethical dilemmas have arisen and are illustrative of the portending future of genomic medicine. Incidental findings from interpretation of the SEQaBOO genome analysis led to obtaining further Institutional Review Board (IRB) approval to inform families of unanticipated research results. IRB approval was given with the request pending assessment of “the need to know” as determined by medical geneticists on the SEQaBOO staff. Although the etiology of many babies’ DHH has been determined, the incidental findings exemplify the complexity and ethical dilemmas that reporting genetic research results may have on the family of a newborn. In addition, such knowledge can be burdensome for the healthcare team, specifically primary care clinicians. These clinicians are responsible for requesting confirmatory testing of research results and may also have limited knowledge about genetic testing and interpretation.

## 5. Lessons Learned: Copy Number Variants and Contiguous Genes

In addition to single nucleotide variants (SNVs), many genes associated with DHH are characterized by well-known etiologic CNVs [26], making important the assessment of the genome sequence for such anomalies. For example, large deletions of both copies of stereocilin (*STRC*) can be etiologic in mild to moderate sensorineural deafness and are responsible for 5.4–16.1% of DHH diagnoses in mixed-ethnicity populations [27]. Due to a segmental duplication that leads to frequent non-allelic homologous recombination in the *STRC* locus on chromosome 15, contiguous deletions of *STRC* and *CATSPER2* genes [27,28] may occur resulting in Deafness Infertility Syndrome (DIS) in males. Informing a family of an infant’s hearing impairment can be valuable for clinical management, but concomitant reporting of the possibility that a male child may have fertility problems in adulthood adds additional distress for the family. SEQaBOO investigators identified a male participant with contiguous deletions of *STRC* and *CATSPER2.* Due to the fact that SEQaBOO is a research study designed to identify genes associated with hearing (and potentially secondary findings as reported in ACMG SF v 3.0), SEQaBOO investigators debated whether to disclose the associated infertility phenotype in a male newborn as the fertility issue would be considered as an adult phenotype. In this case, a decision was made to disclose this finding to the family, in addition to the origin of the DHH, for two reasons: (1) DIS is a recognized heritable deafness syndrome including infertility in males, and (2) the contiguous *STRC/CATSPER2* deletion would be revealed to the family upon clinical validation of the research result.

As part of this research study, all pathogenic CNVs are scored based on the American College of Medical Genetics and Genomics recommendations for reporting pathogenicity of CNVs [29]. Added benefit is derived in reporting CNVs associated with DHH; however, given the complexity of interpretation for genome-wide CNV analysis, there are ethical implications that warrant consideration. For instance, many CNVs detected in this study are not likely to be pathogenic which leaves families without an etiology for their child’s DHH, and may cause a psychosocial burden. Both positive and negative ethical implications arise when determining which CNVs should be reported to families. For example, determining a child’s etiologic DHH diagnosis can influence the future care and management of the DHH, but identifying a variant associated with a later onset disorder can result in a significant burden for the family, clinicians, and healthcare system. These findings present the importance of developing guidelines for reporting CNVs identified in the newborn period as part of a research study, especially when clinical confirmation may be beyond available routine genetic testing. Another ethical aspect to consider is the benefit that reporting this information has for the child. Identifying a child’s predisposition to fertility issues at birth can be the source of psychological distress and subject the child and family to additional stress throughout the child’s life. Thus, it is imperative to respect the child’s autonomy when complex ethical dilemmas arise; this is a challenge that will remain apparent as genomic sequencing technologies and access to such technologies improve, especially in population-based genomic NBS.

## 6. Lessons Learned: Variants of Uncertain Significance and Carrier Status

Genome sequencing can provide useful information that translates into a timely diagnosis, early intervention, and overall improvement of health outcomes. However, genome sequencing presents a level of uncertainty whenever indeterminate results are identified. Variants of uncertain significance (VUS) are genetic variants of uncertain pathogenic potential, as there is insufficient evidence to determine whether the variants cause the phenotype. When clinicians receive such genetic results, additional investigations, such as research and functional studies, clinical correlations, segregation analyses, and case-control statistics, are needed to elucidate or rule out pathogenicity. Further, as broader genetic testing approaches are implemented in medical care, the likelihood of obtaining uncertain results increases. Discussion of testing limitations and the type of genetic results should be included regularly during pre- and post-genetic counseling. Uncertain results may often have psychosocial implications for both patients and clinicians, especially when a VUS is present in a gene, or genes, related to the phenotype. However, as the clinical significance of a VUS is not well established, such variants should not be considered in decisions for clinical management or interventions.

Two potential paths are possible when disclosing VUSs. Some patients experience a positive view or hope that learning indeterminate results most likely indicates that a pathogenic or true diagnosis was not confirmed, whereas others may express adverse emotions, such as distress, frustration, guilt, and confusion as they lack understanding of what a VUS truly means for them or their clinical care [30]. Uncertain results also present ethical dilemmas as they may cause patient dissatisfaction, mistrust of genetic testing, the clinician or science, and uncertain clinical utility, thus complicating the counseling process.

Another source for uncertainty arises when sequencing the genome for DHH-associated genes, as it is not uncommon to identify individuals carrying a single pathogenic genetic variant in a gene associated with recessive DHH (i.e., the individual has a heterozygous variant only in one copy of a gene associated with recessive DHH). Given that up to 80% of DHH is inherited in a recessive pattern [6], there is potential benefit in reporting parental carrier status for the risk associated for future children, whereas reporting carrier status in a newborn provides limited benefit for the family and adds burden and concern. Thus, ethically reporting carrier status in an infant who will not be of reproductive age for over a decade provides minimal beneficence to the family and the child.

## 7. Lessons Learned: Chromosomal Structural Rearrangements

Previous studies highlight the importance of integrating the assessment of chromosome rearrangements involving DHH-related genes into comprehensive genome sequencing; several cases have been detected of a chromosomal translocation was found to disrupting a gene responsible for an individual’s DHH diagnosis [31,32,33,34,35]. Chromosomal structural rearrangements may result in repositioning of segments of chromatin known as translocations, inversions, and duplications/deletions, among others. These events can result in balanced or unbalanced genomes. Balanced translocations are the most frequently reported chromosomal structural rearrangements, estimated at approximately one per 500 individuals [36]. A balanced chromosomal translocation may not involve copy number alterations or gene disruption and, most often, is not responsible for a clinical phenotype. In fact, most balanced translocation carriers are unaware of their chromosomal rearrangement. However, such a genetic alteration may be discovered during childbearing years as individuals with such rearrangements are at increased risk of producing unbalanced gametes. This typically presents with a history of infertility due to recurrent miscarriages, fetal death, or children born with congenital anomalies and/or physical–intellectual disabilities [37]. When a member of a couple harbors a balanced reciprocal translocation, the risk of having an affected child with congenital anomalies ranges from 6 to 12% [36,37]. Dong and co-authors present findings indicating that greater than 11% of couples with recurrent miscarriage are at risk to have a chromosomal abnormality [38]. Thus, balanced translocation information can be informative for reproductive planning and decisions. However, its association with DHH may be etiologic or not yet known.

With the aid of our comprehensive genome analysis tools, the SEQaBOO study has encountered two cases thus far of both an infant and one of their parents being a carrier of a balanced chromosome rearrangement. In one case, it was possible the rearrangement was etiologic for the child’s DHH as a hearing-related gene was disrupted, and the carrier parent also had mild hearing loss. In the other case, the child ultimately passed their audiology exam and did not have DHH, and it appeared unlikely the translocation would cause a phenotype in the carrier (parent or child). The concern then became, not for the proband, but for the young parents who were (possibly unknowingly) at risk of having a child with an unbalanced chromosome complement. Furthermore, the proband would have the same reproductive risk as the parents when of reproductive age. A decision was made to disclose the finding of the translocations to both families, who were aware from an infertility work-up that a member of each couple harbored a rearrangement. Both newborns were conceived by in vitro fertilization (IVF), and no additional genetic testing was performed on the embryos. While reproductive decision making is not included in traditional Wilson–Junger screening criteria, newer criteria frameworks provide insight into the added benefits beyond the individual child, such as family interests, but without ignoring the best interests of the child [39].

## 8. Discussion

Advancements in technologies make possible the application of sequencing in newborns in a timely fashion. This enables interpretation of genetic variants as the molecular etiologies for the clinical phenotype of interest. However, fully informative interpretations of the pathogenicity of variants remains under development, and the possibility of unintended discovery of incidental findings is challenging [40]. The burden and benefits of knowledge obtained from comprehensive newborn sequencing warrants ongoing ethical discussions and prudent considerations.

Genome sequencing is now able to provide information about chromosomal structural rearrangements and occasionally offers clinical utility to identify the etiology of the disorder. It remains important to consider, when involving parents (trio) or single parents (duo), that such rearrangements can be informative for parents themselves, revealing an increased reproductive risk for the couple and biological relatives. Identification of incidental findings in newborns can affect future reproductive outcomes for the family and biological relatives and may be unrelated to the initial indication for genetic testing. In such cases, thoughtful and careful consideration of the utility and importance of reporting incidental findings on newborns for late-onset situations comes with a burden for both the family and clinicians.

The clinical and research team should be aware of some key elements when disclosing incidental findings to families involved in genomic NBS. It is important to contemplate and appreciate the level of distress and anxiety a family may experience when receiving results of incidental findings. This discussion is also impacted by the couple’s level of understanding about genetic information, including different perspectives between the couple themselves. An additional level of complexity is related to the parent’s actionability towards these results, as most disorders with late-onset penetrance would not pose immediate health implications. Physicians’ and/or researchers’ personal biases can impact the perception of genomic incidental results, and awareness of this possibility should be considered when deciding to report such results in the newborn period.

With increased utilization of genetic testing will come complexities of interpretation as researchers and clinicians work together to provide the best care for their research participants or patients. Boundaries need to be delineated with regards to genomic data that clinicians share with patients, especially if the genomic findings are identified as part of a research project.

Increasing access to genome sequencing presents a learning curve in which clinicians, patients, and the public will need to adapt to the ever-changing breadth of knowledge. It remains crucial for clinicians to establish the best practices for reporting genomic results to families to ensure patients’ best understanding. Clinicians are ultimately responsible for communicating genomic results to their patients and should strive to deliver the knowledge in a compassionate manner, such that it reduces excessive distress yet is accurate. This is particularly relevant when it comes with the potential psychosocial complexities of presenting information regarding a newborn’s reproductive future. That being said, it remains important to identify the genetic etiology for a child’s DHH as it may impact management and treatment. However, the need for appropriate genetic counselling will continue to be essential, and experts in the field of genetics must be cognizant of placing the burden of knowledge onto primary care physicians and other specialists.

When considering population-based genome sequencing in the newborn period, equity and access to historically underrepresented populations will need to be carefully guarded as it opens another set of ethical issues. Future projects utilizing genomic NBS must first consider equity and diversity as a priority to provide access to advanced technologies for all families to prevent the further broadening of existing healthcare disparities. Although genome sequencing has become more accessible and more cost effective, limitations persist. For example, the interpretation of genomic results is complicated by the lack of diverse genetic ancestry reference data. Equitable access to genomic technologies is important, as access to these technologies is frequently limited by economic factors as well as additional social determinants of health. Other considerations must be kept in mind when proposing equitable population-based NBS, such as follow-up medical care, further genetic testing, and insurance coverage. Increasing knowledge of the genome and its workings brings the benefit of better health outcomes but is not without additional burden as healthcare systems cautiously learn their way forward.

## 9. Conclusions

Advances in sequencing technologies and analytic pipelines facilitate new discoveries. Limited studies of this nature have made possible identification of unexpected findings in newborn genome sequencing. Researchers and clinicians must work together to establish optimal practices for reporting such information to families. Overall, the benefits of genome sequencing in the newborn period are reflected in advancements in newborn screening technologies. Various considerations with public trust in genomic science will facilitate large-scale implementation.
